# Isolation and Structural Characterization of Natural Deep Eutectic Solvent Lignin from Brewer’s Spent Grains

**DOI:** 10.3390/polym16192791

**Published:** 2024-10-01

**Authors:** Karina Antoun, Malak Tabib, Sarah Joe Salameh, Mohamed Koubaa, Isabelle Ziegler-Devin, Nicolas Brosse, Anissa Khelfa

**Affiliations:** 1 Université de Technologie de Compiègne, ESCOM, TIMR (Integrated Transformations of Renewable Matter), Centre de recherche Royallieu, CS 60 319, 60 203 Compiègne, Cedex, Francemalak.tabib@utc.fr (M.T.); sarah-joe.salameh@utc.fr (S.J.S.); mohamed.koubaa@utc.fr (M.K.); 2 Laboratoire d’Etude et de Recherche sur le Matériau Bois (LERMAB), Faculté des Sciences et Technologies, Université de Lorraine, 54 500 Vandœuvre-lès-Nancy, France; isabelle.ziegler@univ-lorraine.fr (I.Z.-D.); nicolas.brosse@univ-lorraine.fr (N.B.)

**Keywords:** brewer’s spent grains, lignin, purity, NaDES, microwave fractionation, TGA, structural characterization, antioxidant activity

## Abstract

Brewer’s spent grains (BSG) offer valuable opportunities for valorization beyond its conventional use as animal feed. Among its components, lignin—a natural polymer with inherent antioxidant properties—holds significant industrial potential. This work investigates the use of microwave-assisted extraction combined with acidic natural deep eutectic solvents (NaDESs) for efficient lignin recovery, evaluating three different NaDES formulations. The results indicate that choline chloride–lactic acid (ChCl-LA), a NaDES with superior thermal stability as confirmed via thermogravimetric analysis (TGA), is an ideal solvent for lignin extraction at 150 °C and 15 min, achieving a balance of high yield and quality. ChCl-LA also demonstrated good solubility and cell disruption capabilities, while microwaves significantly reduced processing time and severity. Under optimal conditions, i.e., 150 °C, 15 min, in the presence of ChCl-LA NaDES, the extracted lignin achieved a purity of up to 79% and demonstrated an IC50 (inhibitory concentration 50%) of approximately 0.022 mg/L, indicating a relatively strong antioxidant activity. Fourier transform infrared (FTIR) and 2D-HSQC NMR (heteronuclear single quantum coherence nuclear magnetic resonance) spectroscopy confirmed the successful isolation and preservation of its structural integrity. This study highlights the potential of BSG as a valuable lignocellulosic resource and underscores the effectiveness of acidic NaDESs combined with microwave extraction for lignin recovery.

## 1. Introduction

Brewer’s spent grains (BSG) is the primary byproduct of the beer brewing process, constituting approximately 85% of the total byproducts generated during production [1,2,3]. Annually, an estimated 38 million tons of BSG are produced worldwide, highlighting the significant volume and potential of this renewable resource [4]. Given its abundance and underutilization, BSG offers considerable opportunities for valorization, thereby promoting more sustainable and circular practices within the brewing industry.

BSG primarily consists of the insoluble residues of barley and wheat grains post-mashing and is composed of a mixture of cellulose, hemicellulose, lignin, lipids, and various soluble compounds [5,6,7,8]. Despite its nutritional value as animal feed, due to its richness in fibers, crude protein, and minerals, BSG remains underutilized. Its high moisture content poses significant challenges for transportation and storage, as it promotes microbial growth, rendering it an unstable product [9]. The chemical composition of BSG can vary significantly depending on factors such as grain variety, harvest time, and specific brewing conditions, including malting and mashing processes, as well as the type and quality of additives used [10]. Valorizing BSG through innovative recycling and reuse strategies offers substantial benefits for both the beer industry and the environment. According to EUROSTAT, approximately 90 million tons of food are wasted annually in the EU, equating to around 179 kg per person [11]. Despite primarily being used as animal feed or sent to landfills, BSGs are rich in fibers (such as cellulose, arabinoxylan, and lignin), protein, and phenolic compounds, making them stand out among agro-industrial byproducts. This abundance, combined with its high market value and potential as a biomaterial, positions BSG as a promising candidate for various biotechnological applications. These includes green-energy production as a biofuel and bioenergy source [1,12] as well as a source of valuable ingredients for the development of products like xylose, acetic acid, and hydroxycinnamic acids [13]. Additionally, BSG can be used as a substrate for microbial growth and enzyme production, incorporated into papermaking processes, or even used for energy production, for example, in the production of second-generation bioethanol [14,15,16].

Among the valuable components of BSG, lignin is particularly important due to its wide range of potential industrial applications. As the most abundant aromatic biopolymer [17], lignin is a complex aromatic polymer that contributes to the structural integrity of plant cell walls and constitutes a significant fraction of BSG [17,18,19]. Extracting lignin from BSG not only enhances the value of this byproduct but also offers a sustainable source of lignin for various industrial applications. Lignin can be used as a precursor for biofuels, bioplastics, and other high-value chemicals [20]. Its antioxidant, antimicrobial, and UV-blocking properties make it an attractive component for developing advanced materials and pharmaceuticals [21,22,23,24]. Overall, lignin’s biocompatibility, combined with these properties and its photoluminescent characteristics, positions it as a promising candidate for a wide range of innovative applications [25,26,27].

Growing interest in lignin recovery from biomass has driven the development of diverse extraction techniques, including physical, chemical, enzymatic, and innovative fractionation methods. Common approaches involve acid, alkali, organosolv, ionic liquid, enzymatic hydrolysis, and physicochemical methods, aiming to enhance lignin extraction and support the advancement of novel lignin-based applications [28]. In recent years, deep eutectic solvents (DES) have gained significant interest for lignin extraction.

DESs have emerged as sustainable alternatives to organic solvents, offering advantages such as low toxicity, biodegradability, low volatility, non-flammability, and high solvation capacity for a wide range of compounds [29,30]. Compared to ionic liquids (ILs), DESs offer several advantages, including ease of preparation from relatively common, inexpensive, and environmentally friendly components, such as choline chloride (ChCl), glycerol, carbohydrates, urea, polyhydric alcohols, lactic acid, amino acids, and vitamins. DESs consist of a hydrogen bond acceptor (HBA), typically choline chloride due to its cost-effectiveness and biodegradability, combined with one or more hydrogen bond donors (HBDs), including sugars, carboxylic acids, or polyols [31]. Natural deep eutectic solvents (NaDESs), composed of common biological components derived from renewable sources, are considered a green technology due to their biodegradability, low toxicity, renewable origins, and ability to remain liquid at low temperature [31,32]. The NaDES market has expanded rapidly in recent years, as they offer many striking advantages from an environmental and economic point of view, notably in terms of biodegradability, durability, low cost, and ease of preparation [33,34]. Acidic NaDESs, including choline chloride-based NaDESs, are particularly promising in lignin recovery due to their high lignin solubility, achieving extraction yields that are similar to or even higher than those of other organic solvents at relatively low temperatures [35,36,37,38,39]. While extraction times for lignin typically range from 30 min to 6 h regardless of the solvent used, ChCl-based NaDESs generally operate within a temperature range of 80–150 °C. In contrast, alkali extraction methods (which produce commercial Kraft lignin) and organic solvent extraction require temperatures between 160 and 200 °C [40,41,42]. Lignin’s solubility in acidic DESs was greater than in basic DESs due to stronger hydrogen bonding, particularly between the carboxylic acid and chloride ions in ChCl-LA and ChCl-OX (oxalic acid) solvents. Additionally, the carboxyl group (-COOH) can react with the hydroxyl group (-OH) on cellulose to form monoesters or cross-linked diesters, which help prevent cellulose dissolution during acid hydrolysis [39]. Recent studies have demonstrated the versatility of ChCl-based NaDESs in various biomass-processing applications. ChCl-carboxylic acid DES pretreatment has been shown to significantly enhance the breakdown of the lignin–carbohydrate complex, facilitating the separation of lignin with high purity levels, ranging between 93.7% and 96.3% [43]. This solvent system’s hydrogen-bond acidity plays a crucial role in improving the efficiency of the biomass fractionation process, highlighting its potential in biomass pretreatment applications. Furthermore, ChCl-LA as a pretreatment solvent demonstrated strong performance in removing lignin from woody biomass and grasses, achieving lignin removal rates as high as 93.1% [44].

In addition to exploring various solvents, innovative and sustainable heating methods have been investigated. Microwave heating, in particular, has garnered significant attention for its effectiveness in lignin extraction techniques with disrupting plant cell walls, which enhances lignin yields and reduces extraction times [45,46]. Microwaves, as electromagnetic waves, penetrate materials and heat molecules directly through dipolar polarization and ionic conduction mechanisms. This process results in uniform heating throughout the material without requiring direct contact, thereby offering benefits such as shorter extraction times and reduced degradation of bioactive compounds [47,48]. Furthermore, several studies have investigated the potential of integrating DESs with microwave-assisted fractionation of BSG to optimize lignin extraction. Liu et al. (2017) demonstrated that microwave-assisted pretreatment using a ChCl acid DES was highly efficient, removing 80% of lignin from poplar in just 3 min. In contrast, the same DES required 9 h at 110 °C to achieve similar lignin removal when microwave heating was not applied [43]. This significant reduction in reaction time highlights the potential of combining DESs with microwave heating for the efficient and environmentally friendly fractionation of biomass. This method shows great promise for enhancing the sustainability of biomass processing by reducing energy consumption and processing time.

This study aims to investigate the use of microwave heating with NaDES pretreatment for lignin isolation. The novelty of this work is the development of a process, specifically applied to brewer’s spent grains, that combines acidic NaDES with microwave technology for enhanced delignification. This integrated approach offers a more efficient method for lignin isolation from biomass. The extraction was performed under various temperature and time conditions, following an initial step to select the appropriate NaDES. Preliminary characterization of BSG included elemental analysis, as well as the determination of carbohydrate, extractives, lignin, and protein content. The extracted lignin was evaluated for yield and purity and further characterized using FTIR and HSQC NMR spectroscopy to assess its chemical composition and structural features. Additionally, the antioxidant activity of the extracted lignin was assessed by measuring its DPPH scavenging activity, providing valuable insights into the effective separation and high-value utilization of lignin.

## 2. Materials and Methods

### 2.1. Raw Materials and Chemicals

In this study, brewer’s spent grains (BSGs) were kindly supplied by the craft brewery “Saint-Médard” in Compiègne, France. The samples exhibited a moisture content of 75.2 ± 2% and were composed of 90 wt% barley and 10 wt% wheat. Upon receipt, the samples were pressed using a pneumatic pump at 2 bar for 5 min, dried at 75 °C for 48 h until reaching a moisture content of 6.6%, and then ground with a ball mill for 5 min at 30 Hz (Retsch, MM 400, Düsseldorf, Germany). Particle size analysis indicated that the resulting particles ranged from 10 to 100 µm.

Choline chloride (99%) was obtained from Fisher Scientific (Illkirch, France), lactic acid (90%) and oxalic acid (≥99.0%) was obtained from Aldrich (Burlington, VT, USA), and formic acid (98–100%) was from Merck (Darmstadt, Germany). Deuterated dimethyl sulfoxide (DMSO-D6) was obtained from Eurisotop (Saint-Aubin, France), DPPH was from Sigma-Aldrich (St. Louis, MO, USA), potassium bromide IR grade (99+%) was from Thermo Scientific (Berlin, Germany), and sulfuric acid (96%) was from Carlo Erba (Val de Reuil, France).

### 2.2. BSG Characterization

For all characterizations performed, the raw material was ball milled (5 min, 30 Hz) and was considered to be dry. Moisture content was determined by weighing approximately 1 g of wet material in a pre-weighed, dry aluminum cup, followed by drying at 104 °C for 24 h.

To determine extractive content, the milled material was subjected to extraction using a Soxhlet set-up. About 3–4 g of dry BSG were placed in a cellulose thimble above a pre-weighed flask with 300 mL of dichloromethane. The extraction process lasted for 8 h (approximately 20 cycles). After extraction, the solvent was removed using a rotary evaporator, and the extractives were recovered. The flask containing the extractives and BSG was dried at 105 °C for 24 h and then weighed after vacuum cooling.

The lignin content in raw BSG was determined using the Klason lignin quantification. Klason lignin refers to the residue remaining after complete acid hydrolysis of the carbohydrate portion of biomass. The Klason lignin content was determined using a modified gravimetric method based on the national renewable energy laboratory (NREL) protocol and TAPPI Standard Test Method 222 OM-15 [49]. Initially, 1.5 mL of 72% sulfuric acid was added to 175 mg of dry, extractive-free biomass. The mixture was agitated during incubation in a water bath at 30 °C for 1 h then diluted with 42 mL of ultrapure water to 4% sulfuric acid concentration and autoclaved at 120 °C (approximately 1.5 bar pressure) for 1 h. After cooling, the mixture was vacuum filtered through a 1.2 μm glass fiber filter in a ceramic “Gooch” crucible. The crucible with the solid lignin residue (acid-insoluble lignin) was dried at 105 °C for 24 h and weighed after cooling. Klason lignin content was calculated as follows:

(1) $$\mathrm{Klason}\;\mathrm{Lignin}\;(\%)=\frac{\mathrm{mass}\;\mathrm{of}\;\mathrm{lignin}\;\mathrm{residue}\;\left(g\right)}{\mathrm{initial}\;\mathrm{dry}\;\mathrm{mass}\;\mathrm{with}\;\mathrm{extractives}\;\left(g\right)}\times 100$$

The filtrate from the NREL protocol (acid-soluble material) was diluted to 100 mL and further diluted 1000-fold. After filtration, the acid-soluble liquid was analyzed using high-performance anion-exchange chromatography coupled with pulse amperometric detection (HPAEC-PAD) on a Dionex ICS-3000 system equipped with a Dionex CarboPac PA-20 analytical column (3 × 150 mm; Sunnyvale, CA, USA). The chromatographic separation was performed using a stepwise gradient elution with a flow rate of 0.4 mL/min, following the protocol established by the LERMAB laboratory (Paris, France) using the method described by Provost et al. [50]. The standards used for analyses included the following: glucose, xylose, galactose, mannose, rhamnose, and arabinose. The polysaccharide content in the samples was calculated based on the corresponding monosaccharide concentrations. All measurements were conducted in triplicate [50].

Elemental analysis of BSG was conducted to quantify carbon, hydrogen, and nitrogen using a CHNS-O Flash 2000 elemental analyzer (Thermo Fisher Scientific, Quebec City, QC, Canada) [51]. The analysis involved heating the samples to high temperatures to decompose them and measuring the resulting gases. Before analysis, the spent grains were ground and dried to remove moisture. The samples were combusted in pure oxygen, producing gases such as CO, CO_2_, SO_2_, and N_2_, as well as water. The concentrations of these gases were measured to determine the content of C, H, and N in the samples. The results were also used to quantify protein content and were compared to the results obtained using the Kjeldahl method (NF EN ISO 5983-2, using an internal standard procedure, PR-16027, at IMPROVE-institut mutualisé pour les protéines végétales, Dury, France). A conversion factor of 6.25 was applied.

### 2.3. Microwave-Assisted BSG Fractionation Using NaDES

Lignin was extracted using a microwave-assisted fractionation process with an acidic NaDES. Three choline chloride (ChCl)-based NaDESs, each combined with a different hydrogen bond donor (HBD)—lactic acid (LA), oxalic acid (OX), and formic acid (FA)—were selected for this study. The NaDES was prepared by mixing dried ChCl, serving as the hydrogen bond acceptor (HBA), with one of the acids in a 1:2 molar ratio. The mixture was heated in a water bath at 60 °C for 60 min and then stored at room temperature. Thermogravimetric analysis (TGA) was conducted to assess the thermal behavior and stability of the NaDES using a TGA 2 SF apparatus from Mettler Toledo, Columbus, OH, USA. The experiments were performed under an inert nitrogen atmosphere with a gas flow rate of 100 cm^3^/min. Samples weighing 15–18 mg were placed in 70 µL alumina crucibles, and the temperature was increased from room temperature to 900 °C at a constant heating rate of 10 °C/min.

The fractionation process was carried out using an Ethos X 2.45 GHz microwave system (Milestone, Sorisole, Italy). Four grams of ground material were mixed with 20 mL of the NaDES and placed in a borosilicate glass tube reactor within the microwave system. The temperature was raised from room temperature to the desired set point over 2 min, maintained at this temperature (temperature plateau) for the specified duration, and then reduced back to room temperature over 5 min. The microwave power was fixed at 600 W. Two sets of fractionation experiments were conducted: in the first, the plateau temperature was varied (130 °C, 150 °C, and 170 °C) while maintaining the fractionation duration constant at 15 min; in the second, the temperature was fixed at 150 °C, and the duration was varied between 10 and 30 min. Some other extraction conditions were optimized based on preliminary experimental results. After fractionation, a dark brown liquid known as “black liquor” was obtained.

To isolate the dissolved lignin from the black liquor, the NaDES/BSG mixture was treated with ethanol at a 1:2 ratio, stirred for 15 min, and then vacuum filtered. The solid residue, primarily composed of cellulose and hemicellulose, was dried overnight in a fume hood. Lignin was precipitated by adding distilled water at a 1:3 ratio to the filtrate and allowing it to settle for 1 h. The precipitated lignin was then collected after centrifugation (4 °C and 5000 rpm for 20 min), washed with water until achieving a neutral pH, and dried overnight at 60 °C.

### 2.4. Lignin Characterization

The purity of lignin extracted via microwave-assisted fractionation was estimated using the NREL protocol, as previously described for BSG. During the acid hydrolysis step of the NREL protocol, the lignin is divided into acid-insoluble lignin (Klason lignin) and acid-soluble lignin. The Klason lignin portion may also contain ash and protein [52]. During hydrolysis, the polymeric carbohydrates are hydrolyzed into their monomeric forms, which dissolve in the hydrolysis liquid and are predominantly found in the acid-soluble lignin fraction. These monosaccharides are subsequently analyzed via HPAEC-PAD (see Section 2.2).

The antioxidant properties of lignin, primarily due to its phenolic groups, were assessed using a DPPH (2,2-diphenyl-1-picrylhydrazyl) scavenging assay with slight modifications [53]. For this assay, 10 mg of lignin was dissolved in 5 mL of DMSO and vortexed. Various volumes of the lignin solution were then mixed with a DPPH solution (a control solution prepared in ethanol at a concentration of 50 mg/mL) in 5 mL flasks, resulting in final lignin concentrations ranging from 10 to 170 mg/L. After stirring for 1 h at room temperature, absorbance was measured at 520 nm using an Agilent Cary 60 UV-Vis spectrophotometer (Santa Clara, CA, USA).

DPPH scavenging activity was calculated with the following formula:

(2) $$I(\%)=\frac{{\mathrm{Abs}}_{\;\mathrm{control}\;}{-\;\mathrm{Abs}\;}_{\mathrm{lignin}\;}\;}{{\mathrm{Abs}\;}_{\mathrm{control}\;}}\times 100$$

where Abs_DPPH_ is the absorbance of the DPPH solution without lignin, and Abs_lignin_ is the absorbance of the DPPH solution with lignin. The IC50 value, representing the concentration of the compound required to achieve a 50% reduction in DPPH radical activity, was also determined.

Lignin samples were characterized using Fourier transform infrared (FTIR) spectroscopy to identify functional groups specific to lignin. This analysis was conducted using a JASCO FTIR 400 spectrometer (Tokyo, Japan) in transmission mode. To prepare the samples, 10 mg of dried lignin was mixed with 1 g of potassium bromide to form pellets. Spectra were recorded across the wavenumber range of 4000 to 400 cm^−1^ with a resolution of 4 cm^−1^ with 16 scans.

To further investigate the lignin structure and identify aromatic units, 2D-HSQC NMR (heteronuclear single quantum coherence nuclear magnetic resonance) analysis was performed. NMR spectra were acquired using a Bruker AVANCE III 400 MHz spectrometer (Billerica, MA, USA) equipped with a BBFO probe. Approximately 50 mg of each lignin sample was dissolved in 1 mL of DMSO-D6 and homogenized in an ultrasonic bath for 1 h. The solutions were then filtered and transferred to NMR tubes. The HSQC experiments were performed using the standard Bruker “hsqcedetgp” program. Acquisition parameters included a spectral width of 9 to 0 ppm in F2 (^1^H) with 2048 data points and a 1.5 s recycle delay and from 165 to 0 ppm in F1 (^13^C) with 256 increments of 192 scans. The total acquisition time of each sample was 24 h, with the DMSO solvent peak at δ 2.5 ppm used for calibration.

## 3. Results and Discussion

### 3.1. BSG Chemical Composition

Brewer’s spent grains (BSGs), primarily derived from the outer layers of barley grains, exhibit variability in their composition influenced by mashing efficiency and brewing techniques. This complexity is further affected by factors such as cereal type, harvest time, hops variety, and specific brewing methods and conditions, resulting in significant variation in BSG composition across different breweries [5,13,54,55]. Despite these variations, BSG is predominantly a lignocellulosic material, rich in fiber, protein, and extractives. For comparative analysis, data from other studies on the same sample are presented. The BSG from the “Saint-Médard” brewery, with a moisture content of 75.2 ± 2%, shows a chemical composition consistent with the literature (see Table 1) [3,5,56].

BSG is primarily composed of lignin, carbohydrates, protein, lipids, and trace amounts of ash [3,7,56,57,58,59,60]. On a dry weight basis, it contains 6.8 ± 0.7% extractives and 17.5 ± 0.5% protein according to the Kjeldahl method. Klason lignin constitutes 15.8 ± 0.3% of the biomass, with glucose being the predominant monosaccharide at 11.6 ± 0.2%, followed by xylose at 5.3 ± 0.1%. Elemental analysis shows carbon, nitrogen, and hydrogen contents of 44.7 ± 1.5%, 3.2 ± 0.2%, and 6.3 ± 0.2%, respectively, with an estimated protein content of 20% based on elemental data.

Lignin, which constitutes approximately 10–28% of the total dry weight of BSG, is an important component characterized by its polyphenolic macromolecular structure. The phenolic compounds serve as a rich source of natural antioxidants, offering an economical alternative to synthetic antioxidants [54,61,62]. Following lignin quantification in BSG through the acid hydrolysis protocol, monosaccharides present in the acid-soluble fraction were also quantified. As shown in Table 1, and supported by the literature, the predominant monosaccharides in BSG are xylose, glucose, and arabinose, with trace amounts of rhamnose and galactose [63,64].

### 3.2. Thermal Behavior of NaDES

**Figure 1:**
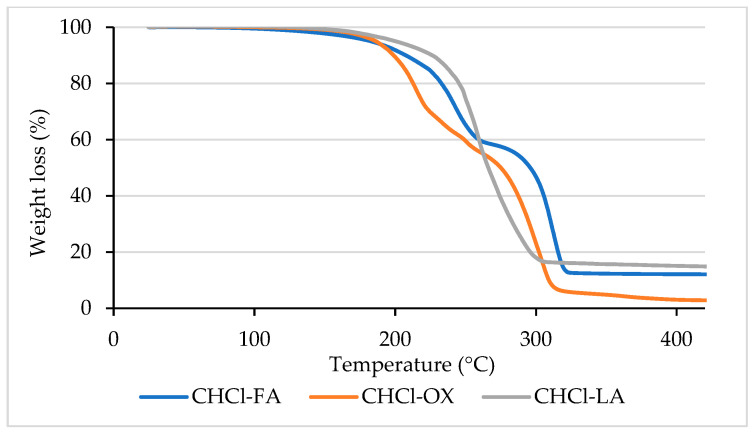
TGA curves of NaDESs used. ChCl-choline chloride, FA-formic acid, OX-oxalic acid, and LA-lactic acid.

Figure 1 presents the TGA data for the NaDES across a temperature range of 100 to 400 °C. The boiling points of formic acid (FA) and lactic acid (LA) are 101 °C and 122 °C, respectively, while the melting point of oxalic acid (OX) is between 189 and 191 °C. Some evaporation occurs at these temperatures with ChCl-FA, ChCl-LA, and ChCl-OX, but no boiling is observed within this range, as indicated by the absence of a rapid decrease in weight percentage. Minimal weight loss was observed for ChCl-OX (~0.9%) and ChCl-LA (~0.6%) up to 150 °C, while ChCl-FA exhibited a slightly higher weight loss (~3%). At 170 °C, the weight loss increased to 2.2% for ChCl-OX, 1.7% for ChCl-LA, and 3.6% for ChCl-FA. The onset decomposition temperature (Tonset) represents the point at which significant decomposition of the DES begins, marking the upper temperature limit to prevent degradation. From the TGA curves, the Tonset values were determined to be 195 °C for ChCl-FA, 188 °C for ChCl-OX, and 228 °C for ChCl-LA. Given that ChCl-LA exhibited the lowest weight loss at elevated temperatures and the highest T_onset_, it demonstrates superior thermal stability. This makes it a promising candidate for biomass delignification processes conducted under our experimental conditions without significant degradation. Thus, ChCl-LA was selected for further experiments.

Tan et al. investigated the effect of functional groups in acid hydrogen bond donors on lignin dissolution within ChCl-based deep eutectic solvents [65]. The study revealed that a short chain with linear monocarboxylic acids (e.g., lactic and formic acids) exhibited superior extraction yields compared to dicarboxylic (e.g., oxalic acid) and tricarboxylic acids, where the additional carboxyl groups hindered extraction due to dimer formation [66]. Alpha-hydroxy acids (ex. lactic acid), with their higher polarity from additional hydroxyl groups, demonstrated enhanced lignin extraction efficiency relative to acids of similar chain lengths and carboxyl group numbers. These acids were more effective than linear and saturated acids. The study by Singh et al. revealed that the combination of lactic acid and choline chloride showed significant lignin solubilization potential; although, it slowed cellulose dissolution. Furthermore, in certain cases, increasing the acid ratio within the solvent mixture further improved lignin dissolution [31].

Other studies have compared different DESs for the extraction of lignin from BSG. In their study, Allegretti et al. studied new protocols for lignin recovery, where choline chloride (ChCl) and betaine glycine (BetG) were used as HBAs, whereas formic acid, acetic acid, and L-lactic acid constituted HBDs. It was shown that choline chloride/lactic acid-based DES was the best in terms of solubilization of the starting biomass, as well as for the handling of the solutions during the DES treatment, and due to the yields of extracted lignin [67].

### 3.3. Lignin Fraction Isolation

#### 3.3.1. Lignin Yield and Purity: Effect of Fractionation Temperature

**Figure 2:**
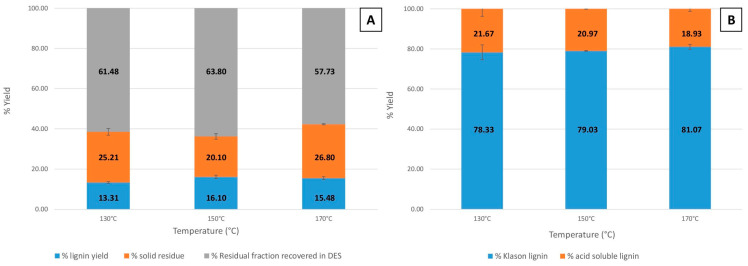
Effect of temperature variation on (**A**) lignin yield and (**B**) lignin purity (Klason lignin).

The lignin yield from microwave-assisted extractions at varying temperatures of 130 °C, 150 °C, and 170 °C is shown in Figure 2A. These extractions were performed using ChCl-LA NaDES as the solvent. The corresponding lignin purity, determined as Klason lignin, for these temperature variations is shown in Figure 2B. In all experiments, the fractionation duration was consistently fixed at 15 min.

It is evident that fractionation temperature significantly influences both lignin yield and purity. As the temperature increased, the lignin yields initially rose from 13.31 ± 0.44% at 130 °C to 16.10 ± 0.85% at 150 °C, followed by a slight decrease to 15.48 ± 0.74% at 170 °C. Similarly, the solid residue yield varied with temperature, decreasing from 25.21 ± 1.68% at 130 °C to 20.10 ± 1.34% at 150 °C, but increasing again to 26.80 ± 0.28% at 170 °C (Figure 2A). The fraction recovered in NaDESs was determined via subtraction. As shown in Figure 2B, lignin purity (Klason lignin) showed only slight variation with increasing temperature, ranging from 78.33 ± 3.71% at 130 °C to 79.03 ± 0.16% at 150 °C and reaching 81.07 ± 1.20% at 170 °C. Despite these minor fluctuations, the purity values achieved are notably high. Considering both the yield and purity of lignin, it can be observed that the amount of pure lignin (calculated as the product of yield and purity) is approximately 10.42 ± 0.60% (representing 65.9% of the initial lignin in BSG) at 130 °C. The original brewer’s spent grain sample was found to contain 15.80 ± 0.29% of lignin, as shown in Table 1. Subsequently, the yield of pure lignin increases to 12.72 ± 0.67% (80.5% of the initial lignin in BSG) for lignin extracted at 150 °C and remains nearly constant at 170 °C with a value of 12.54 ± 0.63% (79.4% of the initial lignin in BSG). This relationship between temperature and pure lignin yield is attributed to the more efficient lignin extraction at elevated temperatures. Temperature plays a crucial role in the lignin extraction process by facilitating the disintegration of cell walls along with the dissolution of lignin within the solvent. Increasing the temperature improves the solvent’s diffusivity into the biomass structure by reducing the hydrogen bonding within the NaDES [68], thereby facilitating the extraction of more lignin from BSG [40]. Additionally, as noted by Mankar et al. [69,70], elevated temperatures reduce the viscosity of NaDESs, which reduces mass transfer limitations and improves the interaction between the NaDES and BSG [71]. The residual carbohydrate yield remained similar across the temperature range when considering the standard deviations, with values of 21.67 ± 3.71% at 130 °C, 20.97 ± 0.16% at 150 °C, and 18.93 ± 1.20% at 170 °C, which corroborates the other results. Based on the observed results and considering the balance between lignin yield, purity, thermal stability, and process energy consumption, 150 °C was identified as the optimal temperature for lignin extraction. This temperature offers a substantial increase in yield while preserving high purity and minimizing the risk of ChCl-LA NaDES thermal degradation.

#### 3.3.2. Lignin Yield and Purity: Effect of Fractionation Duration

In addition to temperature, fractionation duration is a key factor in optimizing the yield and purity of extracted lignin. Following the NREL protocol, the analysis of the chemical composition of the acid-soluble lignin residue and the quantification of carbohydrate provide valuable insights into the fractionation process of BSG. To explore this further, a second series of experiments on BSG microwave fractionation was conducted, varying the duration between 10 and 30 min while maintaining a constant temperature of 150 °C. These experiments aimed to assess the impact of different treatment durations on lignin chemical composition, yield, and purity (see Table 2). The results are presented on an oven-dry weight basis.

Table 2 highlights the effectiveness of microwave fractionation by showing the high purity of extracted lignin and the reduced residual carbohydrate content. The pure lignin yield, calculated as the product of yield and Klason lignin (acid-insoluble lignin), exhibited a pattern similar to that of lignin yield as a function of fractionation duration. Initially, it started at 16.38% at 10 min, decreased to 12.72% at 15 min, then increased to 13.51% at 20 min, peaked at 16.65% at 25 min, and decreased to 14.32% at 30 min. As shown in Table 1, the original brewer’s spent grain sample was determined to contain 15.80% of lignin. For all extracted lignin samples in Table 2, the obtained values of pure lignin yield (considering the lower limit of the standard deviations) varied from 76.28 to 99.64% of the initial lignin in BSG. According to the literature, samples that contain protein can lead to an overestimation of acid-insoluble lignin [52]. This issue is confirmed by our results from the lignin 2D-NMR HSQC characterization, which revealed the presence of protein residues in the extracted lignin (see Section 3.4.3). Meanwhile, the acid-soluble lignin, primarily composed of residual carbohydrate content, showed a significant decline under certain conditions, starting at 5.75% after 10 min and dropping to 1.96% at 30 min. This trend aligns with the decrease in residual sugar content, further supporting the efficiency of the extraction process in isolating pure lignin from BSG. The lignin extracted after 10 min of treatment, despite being present in the same quantity as that obtained after 20 min of fractionation, contained a slightly higher carbohydrates concentrations compared to the lignin extracted at 15, 25, and 30 min. However, the overall carbohydrate content remained low (<0.5 wt%), primarily consisting of sugars associated with cellulose (glucose) [72]. These carbohydrates are likely due to lignin–carbohydrate complexes, which were found to be resistant to cleavage during DES treatment under the specified conditions. It is common for lignin products, particularly those from the papermaking industry, to retain a significant amount of residual carbohydrates or process chemicals [36,73]. Due to the low residual sugar content in lignins extracted with fractionation durations of 15, 25, and 30 min, the initial focus was on the experimental conditions that allowed for the isolation of these lignins. Subsequently, for economic and energy efficiency reasons, the optimal process duration was reduced to a shorter 15 min fractionation time. This study also reveals that the fractionation duration remains significantly shorter than the durations typically observed with conventional heating methods (approximately 24 h). Microwave energy can induce rapid heating, causing the disruption of cell wall structures, the enhancement of solvent penetration, and the breakdown of lignin–carbohydrate bonds, thereby improving lignin isolation [70,71,74,75].

### 3.4. Extracted NaDES-Lignin Characterization

#### 3.4.1. Antioxidant Activity

**Figure 3:**
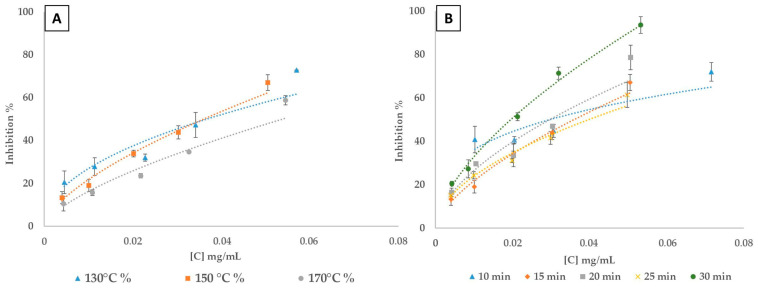
Scavenging activity for extracted lignin for DPPH radical. (**A**) variation of fractionation temperature; (**B**) variation of fractionation time.

The graphs in Figure 3 illustrate the antioxidant activity of lignin extracted under various temperature (Figure 3A) and time (Figure 3B) conditions during the fractionation process. Both graphs show that antioxidant activity, as measured via inhibition percentage, increases with lignin concentration. However, this activity is strongly influenced by the extraction conditions. Lignin’s antioxidant properties stem from its hydroxyl, carbonyl, and carboxyl groups, which neutralize free radicals. By trapping free radicals and by balancing reactive oxygen species to prevent antioxidant-induced oxidative stress, lignin helps stabilize the oxidative environment and reduce cellular damage [76,77].

In Figure 3A, lignin extracted at 150 °C consistently exhibits the highest antioxidant activity across almost all concentrations, followed by lignin extracted at 130 °C, with lignin extracted at 170 °C showing the lowest activity. These findings suggest that 150 °C is the optimal temperature for maximizing antioxidant potential of lignin during extraction. This temperature likely provides the ideal balance between sufficient thermal energy to depolymerize biomass and release phenolic compounds which are responsible for antioxidant properties, without causing significant degradation of the extracted sample. At 170 °C, the elevated thermal energy may induce over-degradation of lignin, leading to the loss of phenolic compounds through condensation reactions or degradation into non-antioxidant byproducts. This is consistent with previous research that indicates the adverse effects of excessive heating on phenolic compounds, reducing their antioxidant efficacy [78]. Figure 3B illustrates that lignin extracted for 30 min (at 150 °C) demonstrates the highest antioxidant activity, with a clear increase in inhibition percentage compared to shorter extraction times. Overall, the results demonstrate that longer extraction times, specifically 30 min, followed by 20 min and 15 min, enhance the DPPH inhibitory effects of the extracted lignin. The lignin sample extracted after 25 min of fractionation displayed a similar, though unexpected behavior to the sample extracted after 15 min of fractionation. Extended extraction durations facilitate more complete depolymerization of the lignocellulosic matrix, enhancing the release of phenolic compounds crucial for antioxidant activity. Shorter extraction times may not allow sufficient time for this breakdown, leading to lower antioxidant activity [79,80]. Lu et al. [81] reported that lignin extracted with acetic acid–water exhibited higher antioxidant activity compared to other lignin samples, such as Kraft lignin and ethanol-extracted lignin. The acetic acid–water lignin had an average IC50 (inhibitory concentration 50%) of approximately 0.7 mg/mL, indicating higher antioxidant activity. In comparison, the IC50 values for Kraft lignin and ethanol lignin were notably higher, at 3 mg/mL and 1.5 mg/mL, respectively. Under our experimental conditions, the optimal conditions for ChCl-LA lignin identified above—treatment at 150 °C for 15, 20, and 30 min—resulted in IC50 values of 0.040, 0.030, and 0.022 mg/mL, respectively. These results show that the antioxidant activity for ChCl-LA-extracted lignin is almost identical across these times and superior to that of Kraft lignin, acetic acid–water lignin, and ethanol-extracted lignin. Therefore, to minimize energy input while optimizing the antioxidant potential of lignin, a temperature of 150 °C and an extraction time of 15 min appear to be ideal for balancing structural breakdown and phenolic content preservation.

#### 3.4.2. FTIR Lignin Characterization

Fourier transform infrared spectroscopy (FTIR) is an essential analytical tool for indicating changes in the composition according to the evaluation of the functional groups belonging to each component.

**Figure 4:**
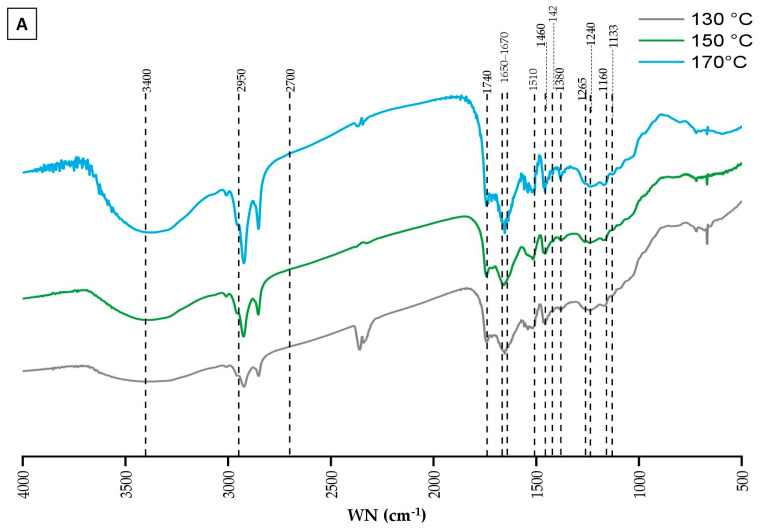

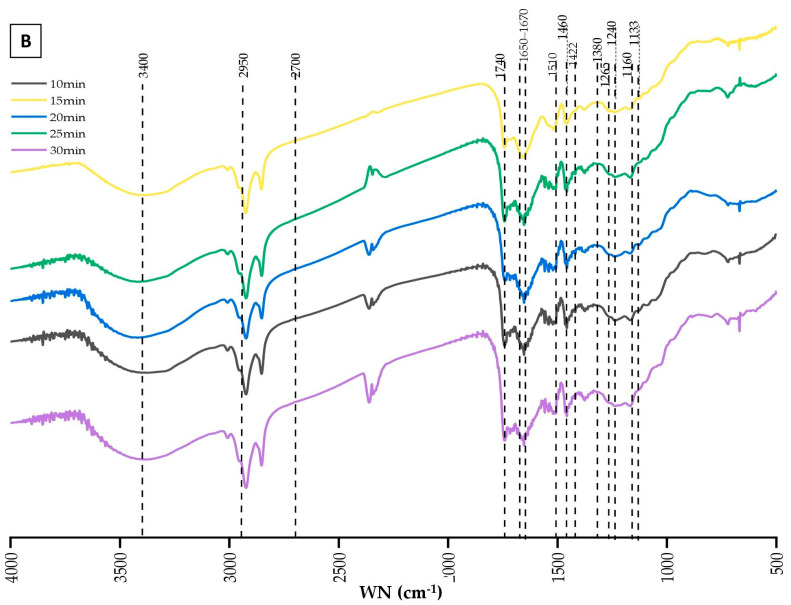
FTIR analysis of extracted lignin. (**A**) variation in fractionation temperature. (**B**) variation in fractionation duration.

Figure 4 presents the FTIR spectra, illustrating the chemical changes in lignin extracted from BSG under varying conditions of temperature (Figure 4A) and reaction duration (Figure 4B). These spectra display the characteristic signal patterns typically associated with lignin allowing for the identification of functional groups through their absorption bands [82]. The spectra were interpreted by correlating the observed wavenumbers with those documented in the literature [83,84,85,86]. Despite overall similarities across the spectra, subtle variations in peak positions and intensities were noted, suggesting minor differences in functional groups or structural features between the lignin fractions. However, the intensities of these absorption bands were not quantitatively compared, as unequal quantities of lignin were analyzed, which could affect relative peak intensities.

In Figure 4A, the analysis of functional groups provides insights into how microwave fractionation temperature influences lignin characteristics. A broad absorption band around 3400 cm^−1^ is observed across all spectra, corresponding to O-H stretching vibrations from hydroxyl groups. This region (3100–3700 cm^−1^) represents a superposition of O-H groups, including phenolic, aliphatic hydroxyls, and free O-H groups [86]. C-H stretching vibrations in methyl and methylene groups are indicated by absorption bands within the range of 2950–2700 cm^−1^, confirmed with bands at 1460 and 1422 cm^−1^, corresponding to CH_3_ bending and CH_2_ scissor vibrations, respectively. The consistency of these bands across different temperatures suggests that these functional groups remain stable under the tested conditions. An absorption band at 1740 cm^−1^, indicative of C=O stretching vibrations in carboxyl and ester groups, suggests that ester linkages between lignin and hemicelluloses were preserved throughout the ChCl-LA NaDES treatments. Additionally, a band at 1650–1670 cm^−1^ indicates the presence of conjugated carbonyl groups, such as α-CO and coniferyl aldehyde groups, within the lignin structure, which are sensitive to structural characteristics and vary depending on the lignin’s nature [86]. The characteristic absorption peaks at 1510 and 1460 cm^−1^ are attributed to the core structure of the aromatic benzene ring and its stretching vibrations, with the peak at 1460 cm^−1^ potentially also relating to methoxyl C-H deformation. The peak at 1380 cm^−1^, corresponding to the C-H vibrations of methyl or methylene groups within the lignin structure, indicates that the fundamental lignin skeleton remains intact after fractionation under these conditions. The intensities of the typical lignin triplet at 1510, 1460, and 1380 cm^−1^ do not show significant variations across different temperatures, suggesting that the aromatic ring structure of lignin remains relatively stable. However, changes in peaks within the 1600–1400 cm^−1^ and 1470–1350 cm^−1^ regions suggest potential structural modifications in the aromatic network due to thermal treatment, possibly involving condensation or fragmentation reactions that alter the lignin’s structural integrity. The peaks around 1265 cm^−1^ and 1240 cm^−1^ (visible in all spectra), attributed to ring breathing of G units and C-O stretching vibrations, remain consistent across temperatures, indicating the persistence of G units despite the thermal treatments [87]. Other S/G-type bands, such as those at 1133 cm^−1^ and 1160 cm^−1^ (in-plane C-H bending), are noticeable in all lignin samples [88]. The 1133 cm^−1^ band is particularly notable as a primary indicator of syringyl structures, associated with the planar bending vibrations of aromatic rings and the C-O bond of the methoxy group.

Figure 4B shows the FTIR spectra of lignin extracted from BSG over varying reaction durations, ranging from 10 to 30 min. The spectra indicate that the overall lignin structure remains stable across different durations, as evidenced by the consistent presence of key functional groups: O-H stretching around 3400 cm^−1^, C-H stretching of aliphatic components within the 2950–2700 cm^−1^ range, C=O stretching vibrations around 1740 cm^−1^, and the aromatic ring vibrations and C-H bending peaks around 1600–1400 cm^−1^ and 1470–1350 cm^−1^.

#### 3.4.3. 2D-HSQC NMR Characterization

**Figure 5:**
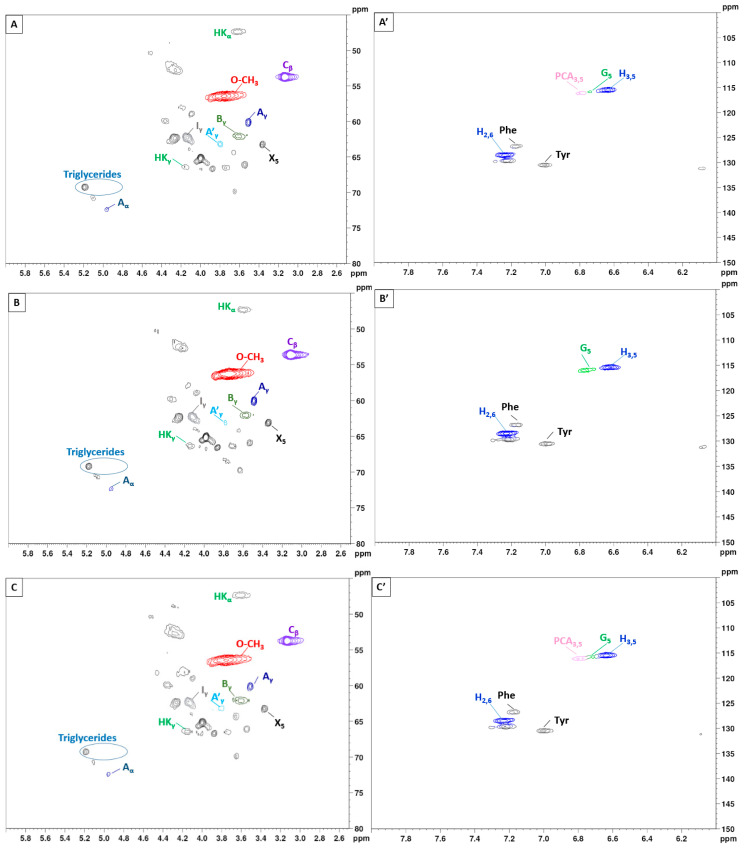
2D-HSQC NMR spectrum of extracted lignin with variation in the temperature; 130 °C, 150 °C, and 170 °C (15 min). (**A**–**C**): side-chain regions: (**A′**–**C′**): aromatic regions.

**Figure 6:**
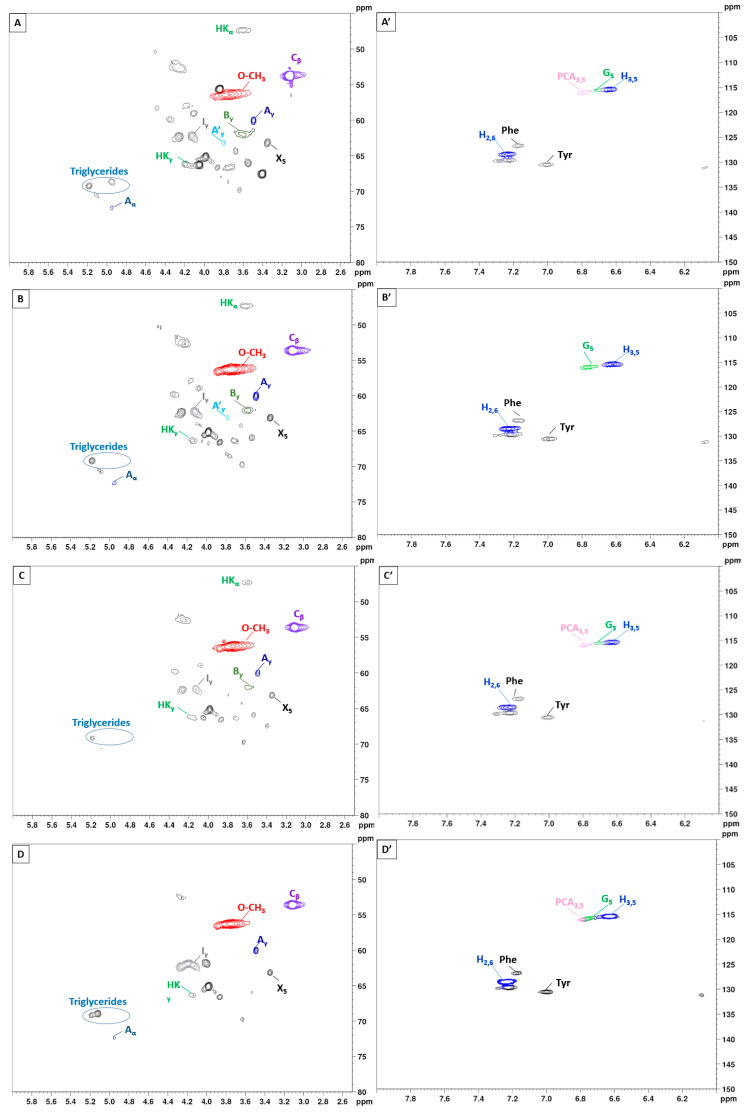

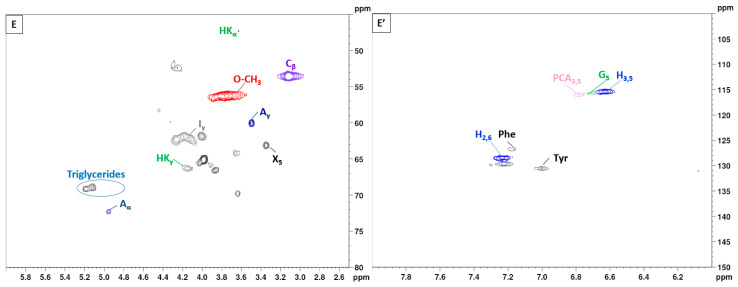
2D-HSQC NMR spectrum of lignin with variation of the extraction duration from 10 to 30 min (150 °C). (**A**–**E**): Side-chain regions: (**A′**–**E′**): Aromatic regions.

**Figure 7:**
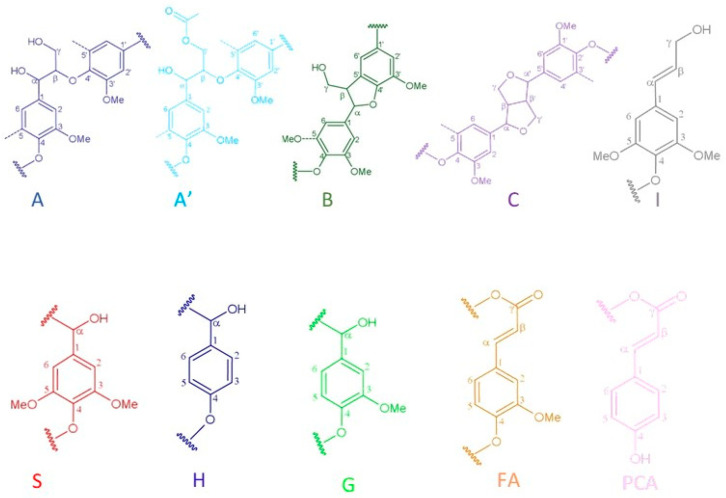
Main lignin substructures cited in the spectra of Figure 5 and Figure 6: (A) β-O-4′ alkyl-aryl ethers; (A′) acetylated β-O-4′ substructures; (B) phenylcoumarans; (C) resinols; (I) p-hydroxycinnamyl alcohol end groups; (S) syringyl units; (H) p-hydroxyphenyl units; (G) guaiacyl units; (FA) formic acid; (PCA) p-coumarates.

To gain further insights into the lignin structure, the extracted samples were analyzed using 2D-NMR. This technique offers details on both interunit linkages and the composition of lignin units. The primary structural components in lignin are in the form of syringyl (S), guaiacyl (G), and p-hydroxyphenyl (H) units, which are interconnected through various aryl ether bonds such as β-O-4′ and carbon–carbon bonds such as β-5′, β-β′ linkages [89,90]. The HSQC NMR spectra, specifically focusing on the side-chain (δC/δH 40.0–80.0/2.5–6.0) and aromatic/unsaturated (δC/δH 100–150/6.0–8.0) regions are illustrated in Figure 5 and Figure 6. Table 3 provides a compilation of the principal lignin cross signals identified in the HSQC spectra, while Figure 7 displays the primary lignin substructures present in the samples.

The side-chain region of the spectra provides crucial insights into the various interunit linkages present in the structure of the lignin polymer. Notably, the spectra reveal prominent signals corresponding to methoxyls, O-CH_3_, (δC/δH 55.6/3.73), and β-O-4′ aryl-ether linkages (substructure A and A′). Although the delignification process has been shown to cause significant cleavage of the β-O-4′ linkage, C–H signals from native β-O-4′ substructures are still evident in all spectra [91]. The C_α_-H_α_ correlations in β-O-4′ substructures were observed at δ_C_/δ_H_ 71.8/4.86 for structures linked to G lignin units. Additionally, the HSQC spectrum shown in Figure 5 reveals signals indicating the presence of acylated γ-carbons (A′_γ_) within the δC/δH range of 63.8/3.83–4.30 ppm, alongside signals for hydroxylated γ-carbons (A_γ_) at δC/δH 59.4/3.40–3.72 ppm [92]. The spectra of BSG lignins (for 10 min at 150 °C and 15 min at 130, 150, and 170 °C) clearly demonstrate slight acylation, occurring exclusively at the γ-position of the lignin side chain. It is well documented that lignins, particularly those from grasses and other herbaceous plants, are naturally acetylated [93,94,95], likely as a structural regulation mechanism at the monolignol level [96]. Moreover, these signals vary with the fractionation severity; they disappear in lignin samples fractionated for 20 to 30 min and are barely detectable in samples fractionated at 170 °C for 15 min. The C–H correlation signals from native C–C linkages, such as β-5’ phenylcoumarans (B_γ_) and β-β’ resinols (C_β_), were also noticeable in Figure 5 and Figure 6. For β-5’ phenylcoumarans, the signals for their C_γ_-H_γ_ correlation are observed at δ_C_/δ_H_ 62.5/3.66 in lignin extracted at 150 °C, for 10 and 15 min, as well as in lignin extracted at 130 and 170 °C, for 15 min. However, this signal diminishes with longer fractionation durations. In contrast, the C_β_-H_β_ correlation for β-β’ resinols (C_β_), identified at δ_C_/δ_H_ 53.5/3.05, is present across all spectra of the extracted lignin and shows minimal variation with changes in fractionation conditions. While phenylcoumarans are susceptible to degradation during delignification processes, resinols are typically more resistant substructures to fractionation conditions [97,98]. Other C–H correlation signals from native lignin linkages such as cinnamyl alcohol end-groups were also observed. Specifically, the C_γ_-H_γ_ correlations in p-hydroxycinnamyl alcohol end groups (I_γ_) were observed at δC/δH 61.4/4.10 in the side-chain region of all spectra. Additional signals from associated carbohydrates could also be found in the aliphatic region of extracted lignin. Indeed, the C_5_-H_5_ correlations from β-D-xylopyranoside units (X_5_) corresponding to residual hemicellulose signals were clearly observed at δ_C_/δ_H_ 62.6/3.40–3.72, confirming the presence of residual carbohydrates on the ChCl-LA NaDES extracted lignins [36,99]. In our case, this signal is very low because of the high purity of extracted lignins (Klason lignin between 74.03 and 87.96%) [36]. A relatively small quantity of Hibbert ketones was identified in Figure 5 and Figure 6, providing further evidence of β-O-4 linkage cleavage via acidic NaDES treatment [36]. The α-protons (HK_α_) were identified by a distinctive peak at δC/δH 47.4/3.62 ppm, while the γ-protons (HK_γ_) were observed at δC/δH 66.2/4.2 ppm [36]. The analyses indicated that the mechanisms of acidic lignin degradation in the NaDES resemble lignin acidolysis catalyzed by HCl [36,100,101,102]. These findings confirm that acidic NaDES treatment can selectively cleave ether bonds, in contrast to alkaline DES treatment [102]. Finally, in all spectra (Figure 5 and Figure 6), signals observed around δ_C_/δ_H_ 69.0/5.15 may come from lipids (triglycerides), which have been detected in some BSG extracted lignins [14].

The primary cross signals in the aromatic/unsaturated region of the HSQC spectra corresponded to the aromatic rings and unsaturated side chains of H, G, and S lignin units, as well as to p-coumarates (PCA) associated with lignin. Figure 5 and Figure 6 indicate that no S units were detected in any of the lignin spectra. Lignin derived from BSG, which originates from herbaceous biomass, contains G, H, and S units. The NMR spectra in Figure 5 and Figure 6 revealed signals in the aromatic region corresponding to the G and H subunits. Specifically, the G lignin units exhibited correlation signals for C_5_-H_5_ at δ_C_/δ_H_ 114.9/6.77, while the H lignin units showed cross signals for C_3,5_-H_3,5_ correlations at δ_C_/δ_H_ 114.5/6.62, overlapping with other lignin signals, and C_2,6_-H_2,6_ correlations at δ_C_/δ_H_ 128.3/7.22, which overlapped with strong signals from proteins. Indeed, the cross peaks observed in the aromatic region of the spectrum are attributed to residual protein units containing phenylalanine (δ_C_/δ_H_ 126.9/7.16) and tyrosine (δ_C_/δ_H_ 130.5/7.0) [102,103]. This finding aligns with the established relatively high protein content in BSG [104]. The absence of the S unit was notable in lignin samples extracted with an acidic NaDES from BSG. However, S units were detected in lignin extracted from BSG using the dioxane/water solvent. Consequently, the extraction of S units could potentially depend on the extractive solvent. Signals corresponding to p-coumarate structures (PCA) were evident in the spectra of all lignins, with cross signals for C_3,5_-H_3,5_ at δ_C_/δ_H_ 115.8/6.83 correlations observed in this region. It has been reported that the cell wall of grasses contains a small amount of ester-linked hydroxycinnamic acid derivatives, such as the phenolic constituents PCA.

The NMR characterization results confirm the purity of the extracted lignin, despite the presence of some residual proteins, sugars, or lipids. Therefore, the ChCl-LA NaDES extracted lignin is suitable for direct use in value-added applications.

## 4. Conclusions

This study demonstrates that microwave-assisted fractionation using acidic NaDESs provides a promising approach for efficient BSG treatment, offering significant reductions in processing time and severity. The ChCl-LA NaDES used in this research demonstrated high solubility and effectively disrupted plant cell structures, facilitating lignin extraction while enhancing both energy efficiency and environmental sustainability. The chemical analysis of BSG aligns with findings from previous studies, revealing a composition primarily composed of carbohydrate, protein, and lignin, along with extractives. The Klason lignin quantification of BSG yielded a value of 15.8 ± 0.3%, which represents the target extraction yield for lignin in this study. Thermogravimetric analysis (TGA) of the NaDESs revealed that ChCl-LA exhibited superior thermal stability compared to ChCl-OX and ChCl-FA, highlighting its suitability as a solvent for lignin extraction under the specific experimental conditions used in this work. The microwave-assisted extraction process, with treatment temperatures of 130, 150, and 170 °C and durations of 10, 15, 20, 25, and 30 min, successfully isolated lignin from BSG. Optimal results were achieved at 150 °C for 15 min, yielding lignin with 80.5% of the initial lignin content from BSG, a purity of 79.03%, and a relatively high antioxidant activity, with an IC50 of 0.022 mg/mL. Additionally, these optimized conditions minimize the risk of thermal degradation to both the solvent and the lignin, thus achieving an ideal balance between yield and quality of extracted lignin. Further characterization of the extracted lignin using FTIR and HSQC NMR techniques provided detailed insights into its structural features and functional groups. FTIR analysis confirmed the presence of essential structural components, with most functional groups remaining stable under the tested conditions. HSQC NMR spectroscopy elucidated specific structural characteristics of lignin and their variations under different extraction conditions. Slight C–H signals from native β-O-4′ substructures remained in all spectra, despite significant cleavage of this linkage during delignification. Other side chain signals (acylated γ-carbons, hydroxylated γ-carbons, and β-5′ phenylcoumarans) varied with fractionation severity. Residual carbohydrate signals (primarily from hemicellulose) were low, indicating high lignin purity with ChCl-LA NaDES extraction. No S units were detected in the lignin spectra, though they were present in lignin extracted from BSG with dioxane/water, suggesting solvent-dependent extraction of S units. Other cross peaks indicated residual proteins (phenylalanine and tyrosine). Overall, this research confirms the viability of using acidic NaDESs, particularly ChCl-LA, for lignin extraction from BSG. These findings highlight the potential of BSG as a valuable source of lignin, with significant implications for its application in various industrial processes.

## Figures and Tables

**Table 1:** Chemical composition of BSG sample.

		BSG Mass Content (%, DRY Weight Basis) ^a^	Zeko-Pivač et al. (%, Dry Weight Basis) [56]	Ribeiro-Sanches et al. (%, Dry Weight Basis) [3]
Extractives		6.8 ± 0.7	nr ^b^	7.99 ± 0.06
Protein (Kjeldahl)		17.5 ± 0.5	20.93 ± 2.38	21.26 ± 0.12
Klason lignin		15.8 ± 0.3	11.41 ± 6.76	16.81 ± 0.14
Monosaccharides	Glucose	21.42 ± 4.81	17.50 ± 0.05	21.7 ± 1.4
	Xylose	nr	24.82 ± 0.29	13.6 ± 0.8
	Rhamnose and arabinose	10.37 ± 10.17	4.81 ± 0.01	5.6 ± 0.4
	Galactose	nr	nr	nr
	Mannose	nr	nr	nr
Elemental analysis	C	nr	nr	nr
	N	nr	nr	nr
	H	nr	nr	nr
Protein (elemental analysis)		20.0	nr	nr

^a^ three replicates. ^b^ not reported.

**Table 2:** Effect of fractionation duration on chemical composition, yield, and purity of extracted lignin samples. The results are the average of three replicates.

	Duration of Microwave-Assisted Fractionation (min)				
	10	15	20	25	30
Lignin yield (%)	22.13 ± 1.13	16.10 ± 0.85	17.51 ± 0.82	19.68 ± 0.78	16.28 ± 0.35
Klason lignin (%)	74.03 ± 1.99	79.03 ± 0.16	77.14 ± 0.12	84.62 ± 3.17	87.96 ± 1.22
Pure lignin yield (%) ^a^	16.38 ± 0.95	12.72 ± 0.67	13.51 ± 0.63	16.65 ± 0.91	14.32 ± 0.37
Acid-soluble lignin (%)	5.75 ± 0.29	3.38 ± 0.18	4.00 ± 0.19	3.03 ± 0.12	1.96 ± 0.04
Glucose (%)	0.20 ± 0.04	0.10 ± 0.01	0.24 ± 0.02	0.09 ± 2.49 × 10^−3^	0.03 ± 0.01
Xylose (%)	0.07 ± 0.01	0.03 ± 2.10 × 10^−3^	0.06 ± 0.01	0.02 ± 1.74 × 10^−3^	<0.01
Rhamnoe and arabinose (%)	0.04 ± 0.01	0.02 ± 1.37 × 10^−3^	0.03 ± 2.10 × 10^−3^	<0.01	<0.01
Galactose (%)	0.05 ± 0.01	0.04 ± 2.48 × 10^−3^	0.04 ± 3.17 × 10^−3^	0.02 ± 4.14 × 10^−3^	<0.01
Mannose (%)	<0.01	<0.01	<0.01	<0.01	<dl ^c^
Carbohydrates (%) ^b^	0.36	0.19	0.37	0.13	0.03

^a^ Calculated as the product of yield and purity (Klason lignin). ^b^ Calculated from the sum of monosaccharides. ^c^ Below the detection limit.

**Table 3:** Assignments of main lignin ^13^C-^1^H correlation signals for the lignin substructures shown in Figure 7 and observed in the lignin 2D-heteronuclear single quantum coherence nuclear magnetic resonance spectra (Figure 5 and Figure 6).

Label	δC/δH	Assignement
OCH_3_	55.6/3.73	C−H in methoxyls
A_γ_	59.4/3.40–3.72	C_γ_−H_γ_ in β-O-4′ substructures (A)
A′_γ_	63.8/3.83–4.30	C_γ_−H_γ_ in γ acetylated β-O-4′ substructures (A′)
B_γ_	62.5/3.66	C_γ_−H_γ_ in β-5′ phenylcoumaran substructures (B)
A_α_	71.8/4.86	C_α_−H_α_ in β-O-4′substructures (A)
I_γ_	61.4/4.10	C_γ_−H_γ_ in p-hydroxycinnamyl alcohol end groups (I)
C_β_	53.5/3.05	C_β_−H_β_ in β-β′resinol substructures (C)
X_5_	62.6/3.40–3.72	C_5_-H_5_ in β-D xylopyranoside
HK_α_	47.4/3.62	α-protons in Hibbert ketone (HK) structure
HK_γ_	66.2/ 4.2	γ-protons in Hibbert ketone (HK) structure
G_5_	114.9/6.77	C_5_−H_5_ in guaiacyl units (G)
H_3,5_	114.5/6.62	C_3,5_−H_3,5_ in p-hydroxyphenyl units (H)
H_2,6_	128.3/7.22	C_2,6_−H_2,6_ in p-hydroxyphenyl units (H)
PCA_3.5_	115.8/6.83	C_3,5_–H_3,5_ in p-coumarate (PCA)
Phe	126.9/7.16	Phenylalanine (residual proteins)
Tyr	130.5/7.0	Tyrosine (residual proteins)

## Data Availability

The original contributions presented in the study are included in the article, further inquiries can be directed to the corresponding author.
