# Correction: Antoun et al. Isolation and Structural Characterization of Natural Deep Eutectic Solvent Lignin from Brewer’s Spent Grains. *Polymers* 2024, *16*, 2791

**DOI:** 10.3390/polym18111305

**Published:** 2026-05-26

**Authors:** Karina Antoun, Malak Tabib, Sarah Joe Salameh, Mohamed Koubaa, Isabelle Ziegler-Devin, Nicolas Brosse, Anissa Khelfa

**Affiliations:** 1 Université de Technologie de Compiègne, Ecole Supérieure de Chimie Organique et Minérale, TIMR (Integrated Transformations of Renewable Matter), Centre de recherche Royallieu, CS 60319, 60203 Compiègne Cedex, France; karina.antoun@crittbois.com (K.A.); malak.tabib@utc.fr (M.T.); sarah-joe.salameh@utc.fr (S.J.S.); mohamed.koubaa@utc.fr (M.K.); 2 Laboratoire d’Etude et de Recherche sur le Matériau Bois (LERMAB), Faculté des Sciences et Technologies, Université de Lorraine, 54 500 Vandœuvre-lès-Nancy, France; isabelle.ziegler@univ-lorraine.fr (I.Z.-D.); nicolas.brosse@univ-lorraine.fr (N.B.)

**Error in Affiliation**

In the published publication [1], there was an error regarding the affiliation for Karina Antoun, Malak Tabib, Sarah Joe Salameh, Mohamed Koubaa and Anissa Khelfa. The updated affiliation should include: Université de Technologie de Compiègne, Ecole Supérieure de Chimie Organique et Minérale, TIMR (Integrated Transformations of Renewable Matter), Centre de recherche Royallieu, CS 60319, 60203 Compiègne Cedex, France. 

**Figure Legend**

In the original publication, there was a mistake in the legend for Figure 7. The abbreviation FA was incorrectly defined as formic acid. The correct legend appears below.

**Figure 7.** Main lignin substructures cited in the spectra of Figure 5 and Figure 6: (A) β-O-4′ alkyl-aryl ethers; (A’) acetylated β-O-4′ substructures; (B) phenylcoumarans; (C) resinols; (I) p-hydroxycinnamyl alcohol end groups; (S) syringyl units; (H) p-hydroxyphenyl units; (G) guaiacyl units; (FAs) ferulates; (PCA) p-coumarates.

**Text Correction**

There was an error in the original publication. Equation (1), used to calculate Klason Lignin (%), contained an incorrect expression.

A correction has been made to Section 2.2, Paragraph 3:

$$\mathrm{Klason}\;\mathrm{Lignin}\;(\%)\;=\;\frac{mass\;of\;lignin\;residue\;(g)}{initial\;dry\;mass\;of\;extractive-free\;BSG\;(g)}\times 100$$

**Error in Table**

In the original publication, there was a mistake in Table 1 as published. Several numerical values were incorrect, and some data were mistakenly reported as “nr” (not reported), although values were available. The corrected Table 1 appears below. 

The authors state that the scientific conclusions are unaffected. This correction was approved by the Academic Editor. The original publication has also been updated.

## Figures and Tables

**Table 1:** Chemical composition of BSG sample.

		BSG Mass Content (%, Dry Weight Basis) ^a^	Zeko-Pivač et al. (%, Dry Weight Basis) [56]	Ribeiro-Sanches et al. (%, Dry Weight Basis) [3]
Extractives		6.8 ± 0.7	nr ^b^	7.99 ± 0.06
Protein (Kjeldahl)		17.5 ± 0.5	20.93 ± 2.38	21.26 ± 0.12
Klason Lignin		15.8 ± 0.3	11.41 ± 6.76	16.81 ± 0.14
Monosaccharides	Glucose	11.6 ± 0.2	17.50 ± 0.05	21.7 ± 1.4
	Xylose	5.3 ± 0.1	24.82 ± 0.29	13.6 ± 0.8
	Rhamnose and Arabinose	2.10 ± 0.04	4.81 ± 0.01	5.6 ± 0.4
	Galactose	0.3 ± 0.1	nr	nr
	Mannose	0.10 ± 0.06	nr	nr
Elemental Analysis	C	44.7 ± 1.5	nr	nr
	N	3.2 ± 0.2	nr	nr
	H	6.3 ± 0.2	nr	nr
Protein (Elemental Analysis)		20.0	nr	nr

^a^ three replicates. ^b^ not reported.
